# NIH Workshop on Clinical Translation of Molecular Imaging Probes and Technology—Meeting Report

**DOI:** 10.1007/s11307-014-0746-z

**Published:** 2014-05-16

**Authors:** Christina H. Liu, Antonio Sastre, Richard Conroy, Belinda Seto, Roderic I. Pettigrew

**Affiliations:** National Institute of Biomedical Imaging and Bioengineering, 6707 Democracy Blvd., Suite 200, Bethesda, MD 20892 USA

**Keywords:** Molecular Imaging probes, Human translation, Disease targets, Regulatory science

## Abstract

A workshop on “Clinical Translation of Molecular Imaging Probes and Technology” was held August 2, 2013 in Bethesda, Maryland, organized and supported by the National Institute of Biomedical Imaging and Bioengineering (NIBIB). This workshop brought together researchers, clinicians, representatives from pharmaceutical companies, molecular probe developers, and regulatory science experts. Attendees met to talk over current challenges in the discovery, validation, and translation of molecular imaging (MI) probes for key clinical applications. Participants also discussed potential strategies to address these challenges. The workshop consisted of 4 sessions, with 14 presentations and 2 panel discussions. Topics of discussion included (1) challenges and opportunities for clinical research and patient care, (2) advances in molecular probe design, (3) current approaches used by industry and pharmaceutical companies, and (4) clinical translation of MI probes. In the presentations and discussions, there were general agreement that while the barriers for validation and translation of MI probes remain high, there are pressing clinical needs and development opportunities for targets in cardiovascular, cancer, endocrine, neurological, and inflammatory diseases. The strengths of different imaging modalities, and the synergy of multimodality imaging, were highlighted. Participants also underscored the continuing need for close interactions and collaborations between academic and industrial partners, and federal agencies in the imaging probe development process.

## Introduction

Molecular imaging plays an important role in advancing knowledge about disease pathophysiology, and diagnostic tools have been developed based on molecular imaging probes and technology. As such, the National Institutes of Health (NIH) has actively supported the development of molecular imaging probes since 2003 with targeted programs under a roadmap initiative and initiatives led by individual institutes. Under the theme “New Pathways to Discovery and Molecular Libraries and Imaging Implementation,” a technology development program was established to encourage the discovery and design of highly specific and highly sensitive molecular imaging probes for basic research and clinical applications. Since then, a plethora of innovative molecular imaging probes have been developed with NIH funding and applied to improve the understanding of disease mechanisms and in some instances to diagnose and track human disease progression. While few probes and techniques have been successfully translated for human application, many have become routine laboratory tools or are being used in the preclinical arena. Challenges remain to demonstrate reliable, reproducible, and quantifiable *in vivo* imaging results that are accepted by the medical community, pharmaceutical industry, and the Food and Drug Administration (FDA) as robust measurements for disease diagnoses, clinical trials outcomes, and drug development. The goal of this workshop was to better understand gaps between clinical needs and probe development, and between regulatory requirements and the reality of clinical translation, including the lack of commercial incentives for the private sector. This report is intended to summarize information presented at the meeting,^1^ focusing on the examples discussed and citations provided by the participants, and on the conclusions of the panel sessions. While the authors have included additional examples to those provided by the participants, the goal of this report is to summarize the outcome of the meeting, not to provide exhaustive reviews for each of the topics mentioned. For additional information on the meeting, interested readers are encouraged to view the recording in the NIH videocast archive^2^ and the NIBIB archive of the conference materials.^3^

## Current Clinical Needs and Challenges

The first session of this workshop focused on high-priority clinical needs that could be addressed by the development of suitable molecular imaging probes. The speakers focused on (1) cardiovascular, (2) endocrine and metabolic, (3) neurologic, and (4) inflammatory diseases. Probe development for cancer was not specifically addressed due to the extensive programs in this area already sponsored by the National Cancer Institute.^4^

### Cardiovascular Diseases

There was a consensus that probes are needed for specific, high-risk patient categories in cardiovascular diseases though image-based population screening is not warranted.

#### Identification of Vulnerable Plaques

Patients with vulnerable coronary and carotid plaques are at risk of acute myocardial infarction and stroke. Currently, carotid atherosclerosis burden is assessed by measuring luminal stenosis. State of the art clinical imaging of the lumen of the coronary arteries include cardiac computer tomographic angiography (CTA) for plaque remodeling [1], 2-deoxy-2-[^18^F]fluoro-d-glucose (^18^F-FDG)-PET for inflamed plaque [2], three-dimensional B-mode ultrasound (US) imaging for vessel wall volume measurements [3], and optical computer tomography (OCT) during coronary angiography for determination of plaque erosion [4]. Several preclinical and proof-of-concept techniques have shown promise in detecting high-risk atherosclerotic plaques such as time-of-flight (TOF) bright-blood magnetic resonance imaging (MRI), gadolinium contrast-enhanced black-blood MRI or ^18^F-FDG-PET/CT [5–7], and contrast-enhanced US using microbubbles [8]. Additional noninvasive and invasive imaging techniques for detecting vulnerable coronary plaques can be found in [9].

The challenge remains in identifying vulnerable plaques in asymptomatic subjects using molecular imaging probes and techniques that will help prevent acute cardiovascular events and reduce current hospital admissions and associated costs. While ultrasound imaging has been used to examine carotid plaque properties, MRI and CT can also detect the presence of plaques, and radiolabeled tracers that target inflammation, thrombosis, and proteolysis may help better characterize atheromatous plaques.

#### Imaging Agents to Improve the Triage of Emergency Department Patients With Chest Pain

Standard emergency department (ED) practice for the triage of chest pain patients involves a 24-h hospital stay for electrocardiography and cardiac troponin evaluation followed by a nuclear stress test (*i.e.*, radionuclide myocardial perfusion imaging, MPI) or CTA [10]. Meta-analysis of patient data has shown that coronary CTA is effective in ruling out the ED patients with acute chest pain [11]. With respect to MPI, imaging tracers that have high myocardial extraction, a short myocardial residence time, and less splanchnic uptake all resolvable in a shorter time period are needed so that physicians can complete an entire stress/rest study in less than 1 h. It was thought by participants that this would have a considerable clinical impact by reducing time and cost in ED’s.

#### Probes for Imaging the Biology of Peripheral Artery Diseases

Peripheral artery disease (PAD) is a clinically neglected condition. Because PAD does not affect only large vessels, molecular imaging probes that provide a way to image the biology of peripheral artery diseases (limb ischemia) may offer an opportunity to develop effective drug therapies as alternatives to surgical revascularization and interventional catheter-based approaches. Current imaging of PAD is aimed at anatomical and flow in large vessels using contrast-enhanced ultrasound (CEUS) [12], contrast-enhanced MRA [13], or CTA [14].

#### Imaging Probes Targeting Myocardial Fibroblasts

Myocardial disease from multiple etiologies result in myocardial fibrosis and lead to heart failure [15]. Because the development of fibrosis is a dynamic process involving myofibroblasts and inflammation, molecular imaging probes targeting markers of these processes may be potentially useful [16, 17]. Specific molecular targets in myofibroblasts amenable to imaging are summarized in [18].

### Metabolic Diseases

Common technical challenges for imaging metabolic diseases include very low concentration of imaging targets from a relatively immature biological palette and dynamic metabolic processes.

#### Imaging Pancreatic Beta Cells

The loss in the function of pancreatic beta cells, followed by subsequent cell death underlies type I diabetes. Therefore, the ability to image mass, function, and inflammation of endogenous beta cells, transplanted islets, and cell-based replacements, as well as to assess the delivery of therapeutic constructs are high-priority areas. This capability would aid the differential diagnosis of diabetes, help design and test prevention strategies and therapies, and help monitor an individual’s response to therapy. Imaging techniques and promising tracers and probes currently available in preclinical setting to imaging pancreatic beta cells mass (BCM) and related metabolic pathways are summarized in [19]; however, many questions need to be addressed before these approaches can be utilized for noninvasive quantification of beta cell mass (BCM) in the native pancreas [20].

#### Imaging Human Brown Adipose Tissue

There are two types of adipose tissue: white and brown adipose tissues. While accumulation of white adipose tissue is associated with obesity, it has been shown that lean men have more brown adipose tissue (BAT) than obese men [21], and the metabolic activities of BAT can be protective against diet-induced obesity [22]. Therefore, noninvasive measurement of mass and metabolic activity can establish a better understanding of BAT distribution and associated metabolic activity in humans. Molecular imaging of BAT using PET, SPECT, CT or MRI, or a combination of these imaging modalities in concomitant with targeting tracers can be used to assess different aspect of BAT metabolic activities [23].

#### Imaging Organ Fibrosis

Nonalcoholic fatty liver disease (NAFLD) can progress in a small fraction of people to cirrhosis and liver cancer. The ability to noninvasively image early inflammation and fibrosis would allow staging of NAFLD with identification of people having an elevated risk of progression. Molecular imaging probes and technology are needed to provide a means to differentiate pathological fibrosis from normal aging and to correlate fibrosis with disease progression, and monitoring response to therapies. Current state of the art imaging capability such as MR elastography [24] is not as likely to be as useful in fibrosis detection for the kidney, prostate, and bladder. An additional confound is that kidney and bladder cells tend to accumulate imaging agent upon excretion, making it difficult to image these tissues with external agents.

### Neurological Diseases

There are a number of neurodegenerative diseases with partially-overlapping symptomatology and underlying molecular pathology. Accurate diagnosis has important implications not only for patient outcomes, but also in avoidance of iatrogenic morbidity, reduction of healthcare costs, and in the correct assignment of patient populations in clinical trials. Development of molecular imaging probes for disease-specific molecular targets can help provide definitive diagnoses. The approval of ^18^F-Florbetapir by the FDA for the diagnosis of Alzheimer’s disease (AD) highlights the need for comparably biomarker-specific agents for the diagnosis of other neurological diseases. This will also facilitate the development of disease-modifying therapies for neurodegenerative and other neural diseases.

#### Imaging Probes Targeting Tau Aggregate Subtypes and α-Synuclein

Endophenotyping of patients early in neurodegenerative dementia is clinically difficult but necessary for the discovery and testing of effective new therapies. There are three pivotal biomarkers associated with various neurodegenerative diseases which include the presence of Aβ-amyloid, tau tangles, and α-synuclein. Currently available PET tracers for molecular endophenotyping of neurodegenerative dementia and movement disorders include ^18^F-FDG for the assessment of cerebral glucose metabolism, [^11^C]-PiB for the fibrillar Aβ-amyloid deposition and [^11^C]-DTBZ for the evaluation of nigrostriatal dopaminergic projection terminals [25–30]. Although ^18^F-Florbetapir for PET imaging of Aβ-amyloid has been approved for the diagnosis of AD, molecular imaging probes for diagnosing other protein deposits associated with various human neurodegenerative diseases are still lacking. Tracers are needed to distinguish among diseases associated with the presence of α-synuclein aggregates (Parkinson’s disease *vs.* multiple system atrophy) and tau tangles (progressive supranuclear palsy *vs.* corticobasal degeneration). Tracers are also needed to distinguish among neurodegenerative dementia subtypes.

#### Imaging Probes to Assess Neuronal Integrity

Although MR diffusion tensor imaging (DTI) can determine regions of compromised white matter tract integrity, there is currently no effective tracer to objectively measure vulnerable neurons. This represents a research opportunity that should advance our understanding of neuronal function loss.

#### Imaging Probes to Assess Endogenous Synaptic Neurotransmitter Levels

Currently available PET probes are used to evaluate the health of endogenous dopamine and opiate systems. For example, we have [^11^C]-raclopride (and others targeting dopamine receptors) which are used to evaluate the health of endogenous dopamine system and [^11^C]-carfentanyl to assess endogenous opiate peptide levels and release [31]. Molecular imaging probes that can measure other neurotransmitters are needed (*e.g.*, glutamate, gamma-aminobutyric acid (GABA), and serotonin).

#### Imaging Probes to Assess Chronic Brain Inflammation

Chronic inflammation of the brain has been implicated in many neurodegenerative diseases [32]. Symptoms include increased blood–brain barrier (BBB) permeability, systemic white blood cell recruitment, and microglial activation (reactive gliosis). Currently available tracers to detect these symptoms include [^68^Ga] EDTA for the BBB disruption [33, 34], [^11^C]-AMT for inflammatory cellular response [35], [^11^C]-deprenyl for reactive astrocytes [36], and [^11^C]-PBR28 for mitochondrial translocator protein (TSPO) associated with oxidative stress status [37].

#### Neuroimaging Associated with Obesity and Diabetes

Chronic metabolic diseases upset energy and homeostasis in the body and the brain; this imbalance is implicated in both disease onset and further altered by disease states. Neuroimaging probes and technologies are needed to assess (1) density and function of hormone receptors in the human brain (*e.g.*, insulin, glucagon, leptin, ghrelin, and sex hormones), (2) altered nutrient-sensing pathways, (3) neurotransporters and receptors in the hypothalamus, (4) active sites of obesity drugs, (5) mechanisms whereby atypical antipsychotics cause obesity, and (6) the relationship between dementia and diabetes.

### Inflammatory Diseases

Because inflammation underlies mechanisms for a number of diseases and clinical conditions, molecular imaging probes that identify targets in this process are likely to have broad applicability. Discussion of this area focused on the widespread importance of inflammatory processes in rheumatology, cancer, cardiovascular diseases, as well as a number of other diseases, and the current paucity of specific imaging probes. One example was given of chemokine receptors that play a fundamental role in the migration of progenitor cells during embryonic development of the cardiovascular, vascular, hematopoietic, and central nervous systems, and are associated with more than 23 types of human cancers. Molecular imaging of antagonists of chemokine receptors could allow for the visualization of specific leukocytes subsets in tissue that could be of value in studying, diagnosing, and treating infectious and inflammatory diseases.

## Advances in Molecular Probe Design

The second session focused on recent developments in molecular imaging probe design that could help address the clinical needs identified in the first session. The speakers focused on technical developments that promise increases in diagnostic sensitivity, or specificity.

### MRI Probes

Molecular imaging with MRI presents special challenges. While biomarkers of interest often are present in 10^−6^ to 10^−12^ molar concentration, the sensitivity of magnetic resonance (MR) contrast agents is in 10^−3^ to 10^−5^ molar concentration range. Hence, signal amplification is required to bridge the gap, and also forms the biggest challenge in MR probe design. Paramagnetic metals such as gadolinium, Gd(III), and iron oxide, Fe(II), are regularly utilized in molecular imaging. Most Gd-based MR contrast agents are small, single ions which produce low signal enhancement. Efforts have been made to compensate such shortcoming by manipulating the rotational correlation or water exchange rate of single or clusters of Gd(III) [38]. Recent development of Gd compounds involves nanoplatforms (*e.g.*, dendrimers, liposomes, polymersomes, micelles, emulsions, carbon, or silica nanoparticles) which can carry high payload of chelated Gd to achieve much-improved relaxivity [39]. Among the techniques for encapsulating Gd ions, the porous vesicle membrane design was shown to facilitate water-exchange rate of Gd(III) and increase the overall relaxivity [40]. Superparamagnetic iron oxide nanoparticles (SPION) contain 6,000 to 9,000 iron per particle and provide strong signal contrast by localized reduction of MR signals [41]. Clustered iron oxide nanoparticles generates more pronounced signal reduction in T2*-weighted images [42].

Another important challenge for MR contrast agent design is to generate bioactivated and theranostic agents that can be used in complex, physiological conditions. Bioactivated MR agents can be utilized to report enzymatic activities [43, 44], gene expression (*e.g.*, β-galactosidase [45], β-glucoronidase, caspases [46], metalloproteinases (MMPs) (*e.g.*, [47, 48])) as well as signal transduction and intracellular messengers such as Ca^2+^ [49] and Zn^2+^ (*e.g.*, [50, 51]). It was observed that currently all MR-based molecular imaging probes are for preclinical and investigational use only.

### PET Probes

Discussion of molecular imaging probes labeled with PET isotopes focused on meeting the needs for neurodegenerative diseases. Specific examples were discussed including Eli Lilly’s florbetapir/Amyvid which was approved by the FDA for clinical imaging of Aβ-amyloid in the US in April of 2012 [52, 53]; GE Health Care and Piramal have submitted new drug application (NDA) to FDA for flutemetamol and florbetaben [54] and a phase III trial is planned by Navidea for AZD4694 in 2014 [54]. The Center for Medicare and Medicaid Services (CMS) recently reviewed Medicare coverage for amyloid β imaging and recommended further study to demonstrate improved patient outcomes following diagnostic imaging or imaging to monitor disease progression. With regard to imaging α-synuclein deposits which are present in neurons of Dementia with Lewy Bodies (DLB) and Parkinson’s disease (PD) patients, the development of selective agents for α-synuclein imaging remains a challenge. PET radioligands needed for additional neurodegenerative proteopathies include agents to detect hyperphosphorylated TDP-43 (associated with amyotrophic lateral sclerosis, ALS), huntingtin (associated with Huntington’s disease), and prions (associated with diseases such as Creutzfeldt-Jakob disease, Kuru, and Alpers’ syndrome).

A currently available radioligand for the detection of CNS inflammation is [^11^C]-PK11195 [55, 56]. PK11195 was first shown to have brain binding capability in 1983 [57]. However, this radioligand has several limitations which include low *in vivo* specific binding, thus signal specificity is low [58]. Several additional TSPO-targeting radioligands developed in the past 10 years have offered incremental improvement in specific binding [59, 60]. There are a limited number of published studies of new TSPO radioligands in clinical populations showing potential in MS evaluations [61, 62]. Possible confounds in the development of TSPO-specific radioligands is the existence of TSPO polymorphism even in the control subjects [63–65]. Possible probe designs for the detection of CNS inflammation may target microglial activation markers such as P2X2, MMP, COS-2, H4 CB2, MAO-B, *etc.*

### Ultrasound Probes

The primary challenge in molecular ultrasound (US) imaging is the image resolution, a spatial scale difference of six orders of magnitude between anatomical resolution (of 10^−3^ m) and molecular resolution (10^−9^ m). Another limitation of molecular ultrasound is that currently available US contrast agents have sizes of the order of 10^−6^ m and are, therefore, largely limited to targets within the vascular compartment. Photoacoustic (PA) imaging has emerged as a promising approach because it offers the penetration depth of US and resolution comparable to optical imaging [66]. Nanoparticle-augmented US/PA imaging can overcome the contrast imaging agent size challenge and has been used for the molecular imaging of cancer [66]. The development of molecular ultrasound imaging requires not only probe design but also hardware and software development [67]. Several companies have made available USPA imaging systems to research communities including Visulasonics, Endra, and iThera Medical. Current preclinical application of USPA imaging probes includes the use of epidermal growth factor receptor (EGFR)-targeting plasmonic gold nanosphere for the detection of EGFR-positive and metastatic tumor cells in mice [68]. USPA imaging can also be used to image lipid and microphages associated with atherosclerosis [69–71]. Using gold nanoparticles and USPA imaging, it is possible to track the proliferation, and differentiation of stem cells is possible [72]. Another photoacoustic contrast agent consists of perfluorocarbon nanodroplets; this agent can be activated by laser to generate signal and enhance image contrast [73]. Molecular US probes have the potential to be “smart” or activatable, biocompatible, nontoxic, and clinically relevant or translatable, and multimodal imaging approaches like magnetomotive US [74–76], and magneto-photo-acoustics [77] are promising.

### Multimodality and Multifunctional Probes

Multimodality agents can provide synergistic information by leveraging the benefits of two or more imaging modalities in serial or concurrent session. Multifunctional agents can, in principle, improve efficiency by enabling detection and treatment in one session. The advantages of generating low molecular weight imaging compounds (<1,000 Da) include (1) established chemistry, (2) easy to characterize, (3) homogenous, (4) easy penetration, and (5) clearer path to clinical use. On the other hand, the advantages of generating more complex multiplexed imaging agents (<100 nm) include higher surface area/volume ratio, flexible platform, potential increase in the therapeutic index, and tunable pharmacokinetics.

Several examples of multimodality and multifunctional probes were presented. One example consisted of the conjugation of a dual-modality (SPECT/NIRF) imaging agent to a prostate-specific membrane antigen (PSMA)-targeting urea, which enables sequential, dual modality imaging of experimental prostate tumors at radiotracer level (1–10 nmol) [78]. A proof-of-concept study demonstrated the feasibility in theranostic imaging of metastatic prostate using a dual-modality (SPECT/CT) nanoplex containing multimodal imaging reporters targeting PMSA. The nanoplex was designed to deliver small interfering RNA (siRNA) along with a prodrug enzyme to PSMA-expressing tumor [79]. Polymeric nanoparticles have the ability to accumulate within the tumor interstitium and have the potential for targeting therapy. Based on this observation, PSMA-targeting, docetaxel (Taxotere)-loaded polymer nanoparticles were shown to have selective cytotoxicity against PSMA-producing cells [80] and cause tumor shrinkage in phase 1 trial [81].

The concept of molecular-genetic imaging was introduced several years ago which allows imaging of gene expression directly or indirectly. For example, *in vivo* mouse imaging of herpes simplex virus type 1 thymidine kinase (HSV1-tk) gene expression was demonstrated using [^124^I]-FIAU [82, 83] with PET. Labeled FIAU with [^125^I] or [^131^I] may have potential to be a theranostic agent. Progression elevated gene-3 promoter (PEG-Prom) can be used to drive imaging reporters to detect micrometastatic disease [84]. Challenges identified by the participants for the complex agents include (1) designing dual PET/MR probes with combined chemistry under solid and solution phases, (2) establishing a useful balance between the different sensitivities of PET and MR agents, and (3) maintaining probe affinity and specificity after labeling with PET and MR signal-generating moieties.

It was concluded during the discussion after the first two sessions that although a number of PET and SPECT probes have been studied in humans, most molecular imaging probes remain in preclinical or limited investigational use. A number of possible reasons for this state of affairs were discussed. While some of the probes have promising specificity, nonspecific binding (especially in the CNS) is in most cases still suboptimal for the challenges of regulatory approval and eventual everyday clinical use. For probes with peripheral targets, accumulation in the liver, kidneys, and suboptimal blood clearance continue to present challenges. Some of the speakers noted that while the probe development community had good communication, there needed to be closer cooperation between academic researchers, clinicians, industry, and regulators, such as the cooperation that led from the discovery of PiB to the eventual development and FDA approval of florbetapir.

A vigorous discussion centered on the possible advantages of multimodality and multifunctional probes. While the panel participants, upon questioning by members of the audience, reiterated the advantages and challenges originally enumerated, no clear consensus emerged in this area. Multimodality probe development has been pursued and continues unabated, but there a most promising translational path was not identified.

## Current Needs and Challenges for Industry

The third session addressed clinical needs from the perspective of the challenges faced during pharmaceutical development. In this context, molecular imaging probes can be used as pharmacokinetic indicators to accelerate the screening of promising drug candidates, or they can be used as surrogate biomarkers for pharmaceutical efficacy.

There are several challenges in the clinical translation of new drugs. For instance, it can cost between $300–400 million and an average of 6.5 years for early research/preclinical testing before an investigational new drug (IND) application is filed. An additional 6–10 years on average and $200–300 million are needed to get a drug through clinical trials [85]. There has been a dramatic increase in attrition rates in phase II and phase III clinical trials [86]. Improving R&D efficiency and productivity depends strongly on reducing phase II and III attrition, hence the design of phase II trial is critical [87]. The reasons for this increased failure rate during pharmaceutical development include (1) lack of efficacy, (2) toxicity, (3) poor drug metabolism and pharmacokinetic profiles, (4) paucity of new drug targets, (5) strategic reasons such as competition and risk/benefit ratios, and finally (6) market failure [88]. PET molecular imaging provides direct quantitative measurement to accelerate drug development. Precision medicine including imaging and genetics can mitigate risks involved in clinical trials. For example, PET imaging is used to perform receptor occupancy studies in the CNS for the reason that receptor occupancy study is the only way to directly confirm target engagement and can help prioritize candidates for advancement to clinic. Pharmaceutical companies also utilize preclinical PET imaging extensively to assess mechanism and therapeutic efficacy, *e.g.*, see [89]. Recently, there is a significant increase in academic/industrial partnership around imaging biomarkers, as well as resources/funding/consortia available for academic-industrial partnerships. Consortia in particular continue to set standard for imaging and optimize preclinical models. In conclusion, PET molecular imaging is the future of drug development which can save both time and money in the selection of targets, confirmation of mechanisms, dosage selection, and the overall evaluation of candidate drugs in their development.

The exploratory IND has provided an efficient mechanism for evaluating investigational radiopharmaceuticals in humans, including the specific disease indication in which the test imaging tracer might be used. An exploratory IND will allow for preliminary human studies in a limited number of subjects to perform within subject comparison of structurally-related radiopharmaceuticals, as well as displacement/occupancy studies. This is yet another way in which imaging agents (typically PET molecularly targeted agents) are routinely utilized by pharmaceutical companies in the development of disease-modifying agents. The imaging agents themselves often remain at the eIND stage, and may not reach full FDA approval as imaging agents for humans.

To collect sufficient human data in clinical studies requires (1) good manufacturing practice (GMP)-certified chemistry production, (2) recruitment of healthy subjects and patients in a dedicated research unit with inpatient facilities, (3) image processing capability, (4) regulatory support for clinical trial, IND and institutional review board (IRB) management, and (5) data/project management support. A multidisciplinary team encompassing experts in chemistry, clinical setup, imaging, data management, and regulatory guidelines is needed to efficiently validate and translate new molecular imaging agents for clinical use but can be difficult to assemble in practice. Two examples were presented to illustrate the steps that Molecular Neuroimaging, LLC undertakes in testing agents for central nervous system diseases, [^18^F]-MNI-659 targeting PDE10A to study Huntington’s disease using PET and [^123^I]-MNI-420 targeting adenosine A2a receptor for CNS diseases using SPECT [90–92].

Given the challenges faced by the pharmaceutical development process, the activities provided by the NCI Experimental Therapeutics (NExT) program^5^ were presented, highlighting support of drug development and registration. The activities currently supported by NExT resources include investigational drugs and biologics; investigational imaging agents, academic, biotechnology and pharmaceutical projects, phase 0, 1 and 2 clinical trials, and high-throughput screening; and hit-to-lead and lead optimization. A particularly important concept supported by NExT is that of “shared” INDs. In “shared” INDs, all but the clinical aspects of the IND application can be “imported” from someone else’s IND by a letter of right of reference. The advantages of shared INDs are to avoid the cost and animal burden of repetitive toxicology studies, to save the work needed to organize and present several sections of an IND, to reduce uncertainty as the result of not knowing what FDA has accepted, and finally to save FDA review time so the review can focus on clinical trials. Imaging probes may potentially be classified as orphan drugs with appropriately strong justifications. FDA offers orphan products grant program for drugs, biologics, medical devices, or medical foods, available to domestic and foreign, public or private, for profit or nonprofit entities.^6^

## Clinical Translation of Molecular Imaging Probes

The final session provided discussion of the regulatory perspective that accompanies clinical translation of molecular imaging probes. The session included an example of successful translation and was accompanied by perspectives from representatives from US Federal Government regulatory agencies.

As an example of the successful “bench to bedside” translation of a molecular imaging probe, participants discussed the process and challenges in getting to first-in-human testing of hyperpolarized (HP) [^13^C]-pyruvate for prostate cancer imaging. It took the University of California at San Francisco (UCSF) researchers 10 years to go from the first technology paper on dynamic nuclear polarization (DNP) to IND approval by the FDA for hyperpolarized [^13^C]-pyruvate and subsequent NIH funding of a phase I clinical trial in prostate cancer patients [93]. Challenges that had to be overcome included the (1) development of a sterile setup to generate and deliver HP [^13^C]-pyruvate reproducibly and efficiently to humans, (2) development of pharmaceutical compounding standard operating procedures for pharmacy technicians to produce doses routinely and in a clean room, (3) creation of a physiologically compatible ^13^C MRI radiofrequency (RF) coil with MR imaging protocol and analysis methods [94, 95], (4) establishment of safety and potential patient benefit for IRB and FDA approval, and (5) coordination of expertise from various disciplines from academia, clinical management, and industry.

The FDA noted that including imaging in a clinical trial can provide assurance that subjects have the defined condition, assess if patients are likely to respond to the investigational drug, and potentially shortening the time needed to demonstrate efficacy of diagnostic or therapeutic drugs for FDA approval. Imaging standards in clinical trials should include standardized acquisition and interpretation to control potential sources of bias and variability and verification of data quality and integrity. To establish efficacy in clinical studies of imaging drugs, the FDA recommends that the anatomic, functional, or physiological measurements using an imaging agent be compared with those of a reference products or procedures or a known true standard. If no standard of truth is available, the FDA recommends that a clinical trial be conducted in order to determine the clinical usefulness. To establish effectiveness of therapeutic drugs using imaging, applicants should consider endpoint meaningfulness, determine applicable imaging standards, and most importantly consult FDA reviewers as early as possible. In sum, the key points for implementing imaging in phase 3 clinical trials are (1) adequate, well-controlled investigations, (2) well-defined and reliable methods of response assessment, (3) imaging standardization, and (4) an endpoint with consideration in meaningfulness continuum (*i.e.*, self-evident/established benefit, reasonably likely to predict benefit, bioactivity/pharmacodynamics).

An important consideration in molecular imaging probe development, following FDA approval, is reimbursement. A representative from CMS raised a number of important points concerning Medicare coverage of diagnostic tests, as they apply to molecular imaging probes: (1) diagnostic tests are rarely therapeutic; (2) diagnostic testing may expose a patient to specific short- or long-term risks; (3) while potential harm may occur, diagnostic tests accrue benefits and can inform downstream clinical management of the patients (4) balance of risk and harm should consider the acuity and severity of the patient’s presentation; (5) avoidance of unnecessary or futile treatment can improve health outcomes; and (6) Medicare does not consider cost in making national coverage decisions. A preferred road to diagnostic coverage should provide adequate evidence that the incremental information obtained by new diagnostic technology, compared to alternatives, changes physician recommendations. In particular, this should result in changes in therapy that improve clinically meaningful health outcomes in Medicare beneficiaries.

## Conclusions

In the presentation and discussion sessions, the workshop participants identified pressing clinical needs and development opportunities for molecular imaging probes in cardiovascular, endocrine, neurologic, and inflammatory diseases, which complement the existing vigorous cancer imaging programs. Conventional nuclear medicine probes as well as novel MRI contrast agents, US and multimodality approaches appear promising for different applications. Industry representatives stressed the need for continued collaboration between academic, government, and industry sectors in these efforts, and noted that molecular imaging probe success should be measured both by the collection of probes that serve to help screen for disease-modifying therapies in preclinical settings, as well as the smaller number that emerge as FDA-approved imaging agents. Participants also observed that standardization and validation of protocols in order to obtain regulatory approval for clinical translation of molecular imaging probes and technology is an achievable and desirable goal.

## Footnotes

1 Meeting presenters: Lynne Johnson, MD, Columbia University/Columbia Presbyterian Hospital; Maren Laughlin, PhD, NIDDK/NIH; Kirk Frey, MD PhD, University of Michigan; Joshua Farber, MD, NIAID/NIH; Thomas Meade, PhD, Northwestern University; Chester Mathis, PhD, University of Pittsburgh; Stanislav Emelianov, PhD, UT Austin; Martin Pomper, MD PhD, Johns Hopkins University; Paula Jacobs, PhD, NCI/NIH; Gilles Tamagnan, PhD, Molecular Neuroimaging, LLC; Kevin Maresca, PhD, Pfizer Inc.; Dan Vigneron, PhD, UC San Francisco; Libero (Louis) Marzella, MD PhD, DMIP/CDER/FDA; Louis Jacques, MD, CMS.

2
http://videocast.nih.gov/summary.asp?live=12925

3
http://www.nibib.nih.gov/news-events/meetings-events/workshop-clinical-translation-molecular-imaging-probes-and-technology

4
http://imaging.cancer.gov/

5
http://next.cancer.gov

6
http://www.fda.gov/orphan
